# The Position of Dentistry in Relation to Medicine

**Published:** 1905-11

**Authors:** 


### Editorial.

### THE POSITION OF DENTISTRY IN RELATION TO MEDICINE.

We are beginning to receive from various sources the results of the dental meetings in the East and the West, from national, State, and local organizations, and the two volumes of the Transactions of the Fourth International Dental Congress. While the proceedings of the less pretentious associations are not all in the hands of readers, and probably will not be until the summer solstice is again upon us, there is sufficient to form an opinion that the papers, as a whole, will average about as usual; some scientifically valuable, but the majority seemingly hurriedly put together to fill a gap in the programme. It is not necessary to criticise this, for it always has been and probably always will be, yet these papers, notwithstanding an ephemeral existence, have a certain value.

The subject of dental education has, as usual, been quite thoroughly threshed out, and it is very evident that no new thought can be forthcoming from that quarter.

We have become accustomed to long and labored articles from the pens of medical graduates on the position of dentistry in its relation to medicine, in which these earnestly endeavor to convince the dental mind that there is but one course to reach perfection in dental practice, and that is through the medical school. These articles would be more convincing if the graduates with the M.D. degree would universally show a superior ability in the work of the modern dentist. The writer has failed to discover this superiority, although he has numerous professional friends in possession of medical diplomas. If a careful examination of results be made, it will be evident that it is rare to find that these are any better qualified to treat pathological conditions, met with in the oral cavity, than those educated in the higher dental schools of the present time. It seems a waste of time to argue this question upon theoretical grounds when the results are all around us.

While we have had much from the medical side, and mainly partisan in character, it is not often found that a D.D.S. will present the question so decidedly one-sided as does Dr. Carlton on another page. His argument contains nothing specially new, for, as before stated, there is nothing new to be written upon this well-worn theme; yet, while this is true, his paper is of special note, as it is the product of one of the most cultivated members of our profession on the Pacific coast. It is, however, with some feelings of regret that he has been willing to lend his mental force in what seems to be in the direction of lowering the professional standing of dentistry. The trend of the article is evidently to make the D.D.S. degree a synonym of partial culture, but it is practically no culture at all, for he says, "Medicine and dentistry, though cognate sciences, have always been separately studied. This dissociation is now seen to be a great error, due not alone to the fact that the relation of the mouth and teeth to the rest of the anatomy was not understood, but also because only the mechanical aspect probably appealed to those who first considered the matter. . . . This conception of dentistry as an entity and not as an integral part of medicine has made it an art, possibly a trade, rather than a science." And further, he quotes from another that "The knowledge of life and disease represented by the average D.D.S. degree is certainly deplorably deficient." We are accustomed to this kind of talk from the self-appointed critics of the medical school, and may have the privilege of estimating it at its real worth, but it is not pleasant to have this kind of thought spread upon the pages of a medical journal, and that from the pen of a D.D.S. If it were absolutely true, it would be proper to let all the world know of it, but as it stands it creates the idea that the better class of dentists are ashamed of their calling, because it is not an "integral part of medicine." Its tendency must, therefore, be to give strength to the prejudice existing in the mind of the average medical practitioner against dentistry and dental schools.

Those who have entered dentistry in recent years have but a limited conception of the struggles made to overcome this unjust depreciation of it by medical men. It began openly when Harris failed to convince the medical thought of his day that dentistry was worthy of acceptance as a distinct part of the healing art. He was rejected then, although an M.D., and the D.D.S. has been rejected ever since.

When the amount of superior culture obtainable in that day by the student of medicine is considered, the claim to superiority becomes farcical. It is a matter of medical history, in comparatively recent years, that medical science embraced certain foundation studies, with certain others deemed of vital importance to-day omitted, because practically unknown. The man of less than forty years in the past took his degree in two years of six months, and was not aware that histology was vitally important to the study of general anatomy, or that bacteriology had any close relation to pathological conditions. The question of preliminary education was not considered an important factor, and it is doubtful whether it was regarded as essential, although the advanced social position accorded the medical practitioner drew a larger proportion of educated men to the medical schools.

The higher dental schools of the present time are not only better equipped for the teaching of all the foundation branches of medicine, but the subjects, with the exception of anatomy, are far more thoroughly taught than they were in the medical schools of 1870. This statement cannot be successfully controverted. Not only are these more thoroughly acquired through individual teaching, but instead of two years and sessions of six months, the dentist of the present must take a course of three years of eight to nine months' sessions each. It is true that there are dental schools of a high order and schools that may be termed mediocre, but the same may be said of medical colleges which are turning out yearly imperfectly trained medical practitioners.

The question of what is the best course to pursue in dental schools has not as yet been satisfactorily answered. The practical man has long since recognized that the study of medicine, following the scholastic period, carries the youth beyond the time to begin mechanical training; that the head at this period is being cultivated at the expense of the hands. The other side deems this of no importance, and assumes that the mechanical, including operative, can be performed by specialists, who deserve, from their trade affiliations, no claim to professional consideration. The true dentist of the future is, in their estimation, one who knows nothing of these, but much of so-called medical science, and he will be the oral pathologist of the future. This may possibly come true in the generations yet to be, but what kind of dentistry will that be that knows nothing of that mechanical skill that preserves the natural organs, or substitutes when these are destroyed by age or disease. In a word, the great benefit that has accrued to humanity through dental mechanics would have been lost. Fortunately this is an impossible supposition.

What may be the developments in the future, or the place which dentistry will occupy, may not be possible to foresee, for the problem is one incapable of solution, but dental educators are trying to lay a sure foundation that it is hoped will lead to a system of practice that will measurably be free from criticism.

It must be evident to the careful observer that advanced training has its drawbacks so far as dentistry is concerned. It is unnecessary to dwell upon these, but the man who has graduated in medicine subsequent to his dental college training seems to lack something that the D.D.S. alone possesses. He may have all the skill requisite, and certainly he possesses superior knowledge, for he has spent some six years in obtaining it, but he appears to have come to occupy the position of being neither a good medical man on the one hand, or a happy member of the dental fraternity on the other.

This difficult question will probably be settled in the future by the dental colleges being brought in closer affiliation with the university system of the United States, and then, as now, the dentist will be trained in medicine and dentistry during the undergraduate course of five years' duration. He may carry then the M.D. as a badge of higher professional culture, but the world will know him as a dentist, and not as a stomatologist or as a practitioner of the healing art. It is not believed by the writer that his standing then will be regarded as being equal to the mother from which he sprang. It is a safe rule to always calculate that the man who works with his hands will always occupy a lower level than the individual who labors with his brains, and the dentist of the future must rest content with the conviction that his work, properly conducted, has a value far superior to the average medical practitioner, for the certainty in results of the one and the uncertainty of the latter will in time come to be understood better than it is to-day, and then the practitioner of the healing art will be judged more directly by results obtained.

